# Self-jumping Mechanism of Melting Frost on Superhydrophobic Surfaces

**DOI:** 10.1038/s41598-017-15130-0

**Published:** 2017-11-07

**Authors:** Xiaolin Liu, Huawei Chen, Zehui Zhao, Yamei Wang, Hong Liu, Deyuan Zhang

**Affiliations:** School of Mechanical Engineering and Automation, Beihang University, Beijing, 100191 China

## Abstract

Frost accretion on surfaces may cause severe problems and the high-efficiency defrosting methods are still urgently needed in many application fields like heat transfer, optical and electric power system, etc. In this study, a nano-needle superhydrophobic surface is prepared and the frosting/defrosting experiments are conducted on it. Three steps are found in the defrosting process: melting frost shrinking and splitting, instantaneous self-triggered deforming followed by deformation-induced movements (namely, *in-situ* shaking, rotating, rolling, and self-jumping). The self-jumping performance of the melting frost is extremely fascinating and worth studying due to its capability of evidently shortening the defrosting process and reducing (even avoiding) residual droplets after defrosting. The study on the melting frost self-jumping phenomena demonstrates that the kinetic energy transformed from instantaneous superficial area change in self-triggered deforming step is the intrinsic reason for various melting frost self-propelled movements, and when the transformed energy reaches a certain amount, the self-jumping phenomena occur. And some facilitating conditions for melting frost self-jumping phenomena are also discussed. This work will provide an efficient way for defrosting or an inspiration for further research on defrosting.

## Introduction

Frost accretion on surfaces may cause well-known problems, like reducing visibility of glasses, decreasing heat transfer efficiency of air conditioners, and causing accidents of electric power systems^1,2^. Researchers have made many efforts to design functional surfaces that can delay frost accretion in cold and humid environment or surfaces for defrosting applications^3–7^. Superhydrophobic surfaces are still the most popular candidate for anti-frosting and defrosting, owing to their excellent water repellency^8,9^.

However, the frosting and defrosting processes are quite complicated and are influenced by many factors including humidity, temperature and surface structure. Studying the dynamic process of frost forming and melting on superhydrophobic surfaces is still imperative for understanding the mechanism of frosting and defrosting. Over the years, delaying frost formation on various superhydrophobic surfaces has been widely studied^10–18^. However, the frost propagation is still inescapable even on superhydrophobic surfaces. Under such circumstances, high-efficiency defrosting surfaces that can evidently reduce the energy consumption or residual droplets in defrosting process are still urgently needed. So it is essential to investigate the defrosting process, namely, melting process of the frost on surfaces for developing effective defrosting surfaces.

Recently, researchers have paid more attention to the dynamic performance of melting frost on superhydrophobic surfaces^19,20^. Boreyko *et al*. investigated the dynamic removal of the melting frost on nanostructured superhydrophobic surface and found spontaneous dewetting performance followed by gravitational mobilization^8^. Wang *et al*. reported continuous self-jumping phenomena of melting frost on the superhydrophobic coating, and regarded that the sudden-increased pressure under the frost crystal provided the propelling force. However, in order to achieve the local high-pressure under the frost crystal, they managed to rapidly elevate the temperature of substrate (5 °C/s)^21^. Chu *et al*. conducted defrosting experiments on ultra-slippery superhydrophobic surfaces and found self-propelled melting frost rotating, jumping and sliding^22^. They well analysed the two-dimensional rotating mechanism from the point of energy, but the three-dimensional motion (i.e., self-jumping) mechanism remained unrevealed. From a practical perspective, the melting frost self-jumping is much more attractive and energy-saving due to its capability of reducing the frost contact time and the residual droplets on the surface. From this point, we prepared a nano-needle superhydrophobic surface and studied the dynamic process of the frost melting on it, in order to get deeper understanding of high-efficiency defrosting and its applications.

## Results and Discussion

**Figure 1:**
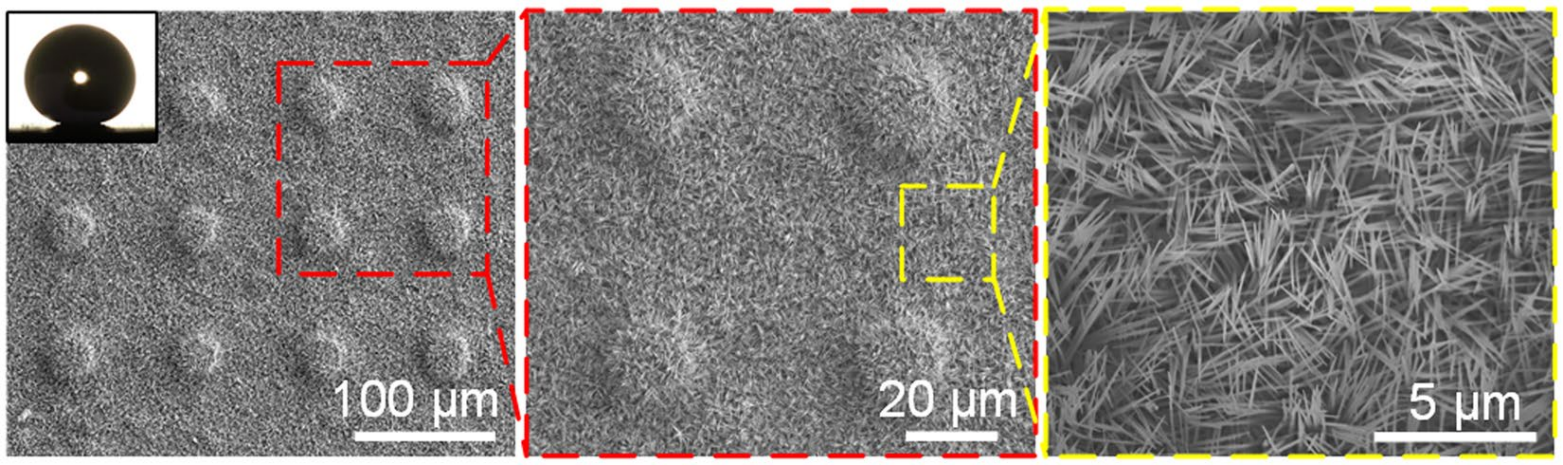
SEM images of the prepared nano-needle superhydrophobic surface with different magnification. The diameter and the height of the pillar are ~40 µm and ~10 µm, respectively. And the central distance of the adjacent pillars is ~100 µm. The inset shows the contact angle of a 10 µL sessile water droplet on the prepared surface.

The nano-needle superhydrophobic surface with micro-pillar (named NNS) was prepared by an electrochemical method (see the Methods section). The SEM images of the prepared nano-needle superhydrophobic surface with different magnification are shown in Fig. 1. The as-prepared nano-needle superhydrophobic surface displayed excellent superhydrophobicity, with an apparent contact angle of 170.0 ± 1.0° and a contact angle hysteresis of 0.9 ± 0.5°. And the advancing and receding apparent contact angles were 170.3 ± 0.8° and 169.4 ± 1.0°, respectively (see Supplementary Fig. S1).

### Frost propagation

In the frosting process, the samples were cooled to −10 ± 1 °C and the humidity of the experiment chamber was controlled to 85 ± 5%. As the sample was cooled down, the water droplets condensed and coalesced on the nano-needle superhydrophobic surface. Previous studies have demonstrated the self-propelled jumping of coalesced water droplets^23–26^. Dropwise condensation and self-propelled jumping of droplets lowered the droplets coverage, and further delayed the ice bridge formation^20,27–29^.

Although the superhydrophobic surfaces showed anti-frosting performance, the condensed water droplets still started freezing at the edges or defects of the samples, owing to the smaller energy barrier for heterogeneous nucleation at substrate edges^9,30^. The freezing line propagated from frozen droplets to the whole surface in virtue of the ice bridge^9^. In this condition, the NNS sample surface was fully covered by frost in 1,980 s, showing excellent frost-delaying performance (see Supplementary Fig. S2).

### Melting frost dynamics in defrosting process

**Figure 2:**
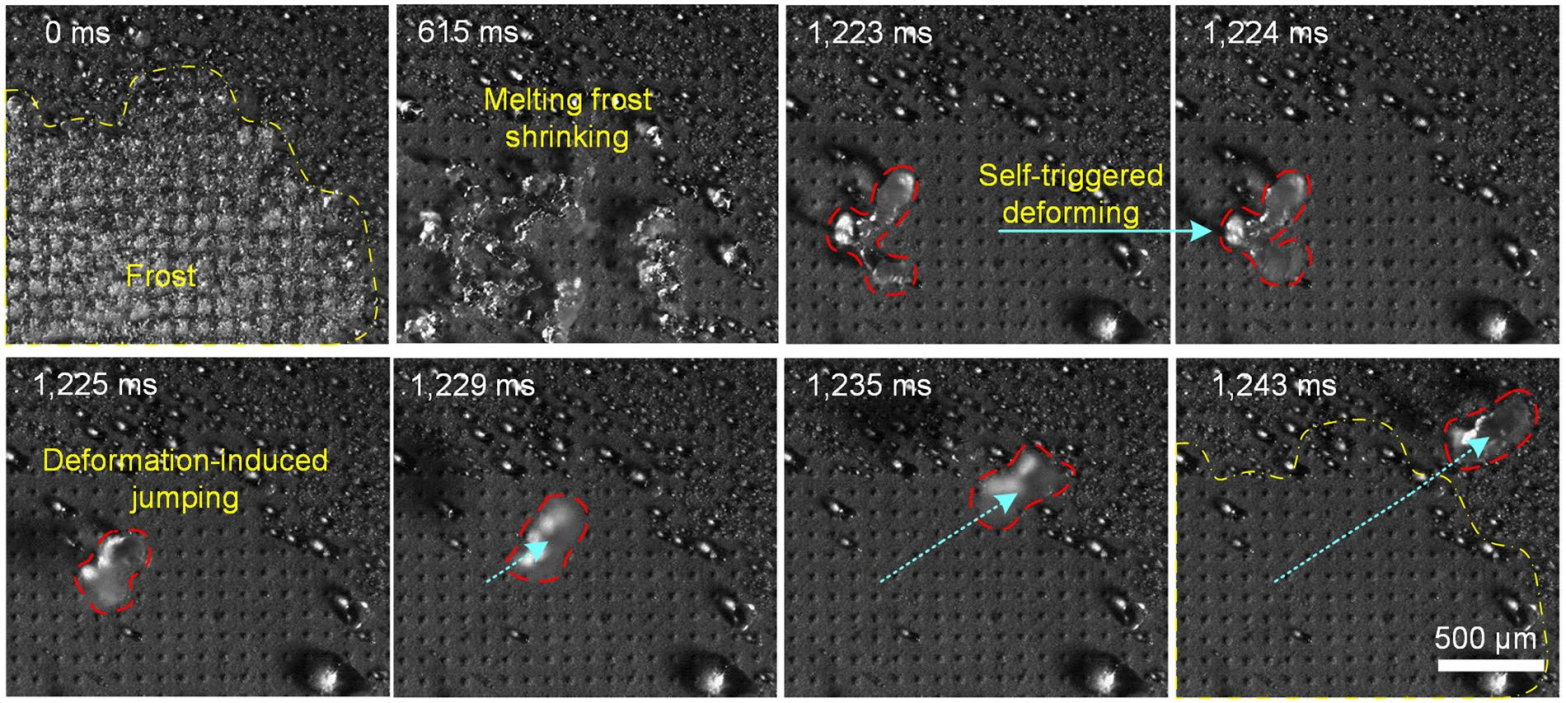
Three steps of the defrosting process—melting frost shrinking and splitting, self-triggered deforming and deformation-induced jumping. The self-triggered deformation finished in less than 1 ms, followed by self-jumping performance of the melting frost.

When the frost was accreted for 10 minutes on the NNS surface, the cooling stage was powered off, and the NNS sample was naturally warmed up to the room temperature by the air. As shown in Fig. 2 (see also Supplementary Video S1), the defrosting process could be mainly divided into three steps: melting frost shrinking and splitting, instantaneous self-triggered deforming and deformation-induced jumping. Existing as frost initially, the solid-state water showed very large superficial area. With the rising of temperature, the frost in the field-of-view started melting and shrinking, leading to a great (but relatively slow) change of superficial area. The released surface energy transformed to kinetic energy, causing mild movements like twisting, sliding or rolling. But no obvious self-jumping of melting frost could be found before it split into small parts. Then the melting frost split into some small irregular-shaped parts and the self-jumping phenomena occurred. Large numbers of experiments demonstrated that the self-jumping of melting frost was always accompanied by great instantaneous self-triggered deformation. Not like shrinking, the self-triggered deformation finished in a very short moment, causing great superficial area change, leading to large amount of energy transformed into kinetic energy. It is also worth noting that, after the self-jumping of melting frost, the frost covered area became very dry and nearly free of residual water droplets (circled by the yellow dash line). Based on Varanasi’s report, the frost accumulated on the surface under such frosting conditions was still in a Cassie state due to the nano-textured structure (e.g., nano-needle structure in our work) of the prepared surface^31^. The extremely low frost adhesion makes it easier for melting frost to detach the surface. With the help of melting frost self-jumping performance, the sample surface can evidently shorten the defrosting process and reduce, even avoid residual droplets after defrosting (see Supplementary Fig. S3).

**Figure 3:**
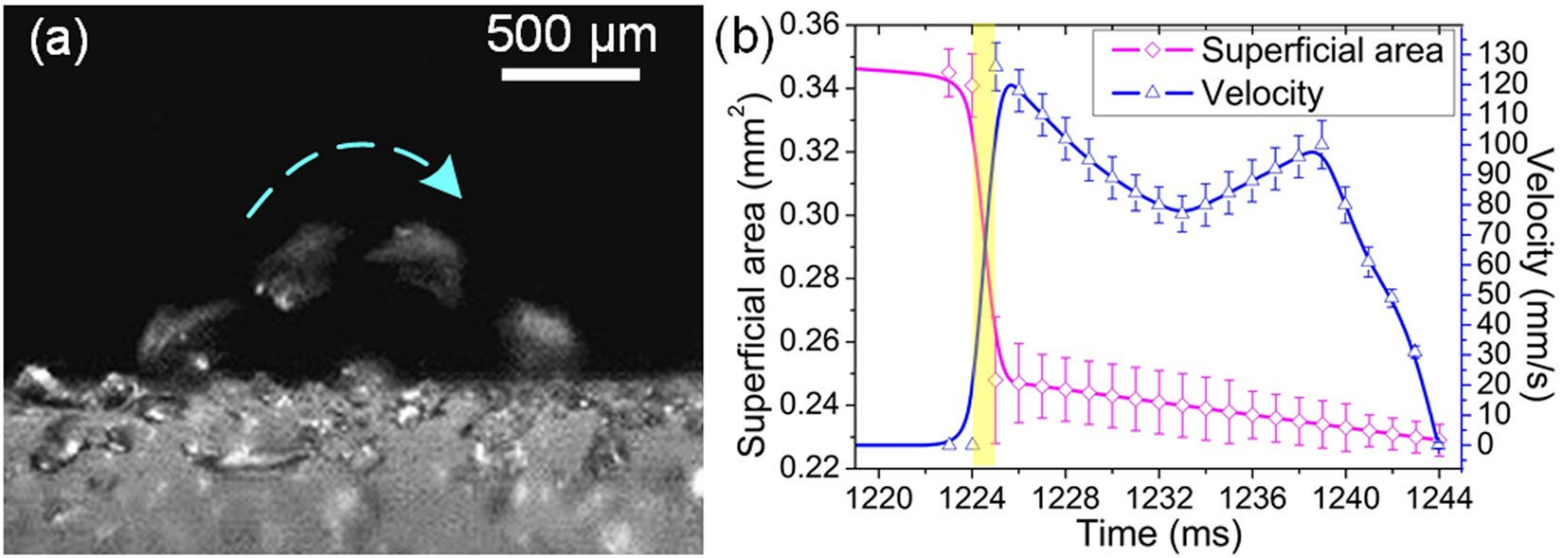
(**a**) Side-view of the melting frost jumping. (**b**) The synchronous change of superficial area and velocity of the melting frost (circled by red dash line in Fig. 2), as a function of time. At 1,224 ms, a great decrease of superficial area and a great increase of velocity occurred at the same moment.

The side-view of melting frost jumping status is also shown in Fig. 3a, and the synchronization of superficial area change and velocity change of the melting frost (circled by red dash line in Fig. 2) is shown in Fig. 3b. The superficial area of the melting frost was roughly estimated by three-dimensional reconstruction using a three-dimensional modeling software “Solidworks 2013” (see Supplementary Fig. S4). At 1,224 ms, a great decrease of superficial area and a great increase of velocity occurred at the same moment. After this moment, the superficial area decreased tardily due to water evaporation, and the velocity changed like a projectile motion.

From the energy standpoint, the surface energy released from instantaneous superficial area change (∆*E*_released_) overcame adhesion loss (∆*E*_adhesion_) and other loss (∆*E*_other_) like dissipation caused by violent droplet oscillations and viscous effect, and the energy left transformed into kinetic energy (∆*E*_kinetic_):

1 $${\rm{\Delta }}{E}_{{\rm{released}}}=\sigma \cdot {\rm{\Delta }}S$$

where *σ* is the surface tension and ∆*S* is the superficial area change;

2 $${\rm{\Delta }}{E}_{{\rm{kinetic}}}={\rm{\Delta }}{E}_{{\rm{released}}}-{\rm{\Delta }}{E}_{{\rm{adhesion}}}-{\rm{\Delta }}{E}_{{\rm{other}}}=0.5\rho \,V\,{\rm{\Delta }}{v}^{2}$$

where *ρ*, *V* and ∆*v* are the density, volume and initial velocity of the melting frost, respectively.

If all of the released surface energy was transformed to translational kinetic energy, the theoretical initial velocity of the melting frost (∆*v*_t_) in Fig. 2 could reach 1.18 ms^−1^. However, the measured initial velocity of this melting frost (∆*v*_m_) just reaches 0.13 ms^−1^, with a transforming efficiency (∆*v*_m_/∆*v*_t_) of 11.0%. And a group of experiments show that the transforming efficiency (∆*v*_m_/∆*v*_t_) of these melting frost is within the range of 10.0~14.0%. Previous research has also demonstrated that the transforming efficiency of two self-propelled dropwise condensate drops is approximately under 17%^23^. In fact, such micro-scale droplets actually exhibit a very low Ohnesorge number where the capillary–inertial effects play a dominant role, and the violent droplet oscillations cause great energy dissipation, leading to a low efficiency of velocity transforming^32^.

**Figure 4:**
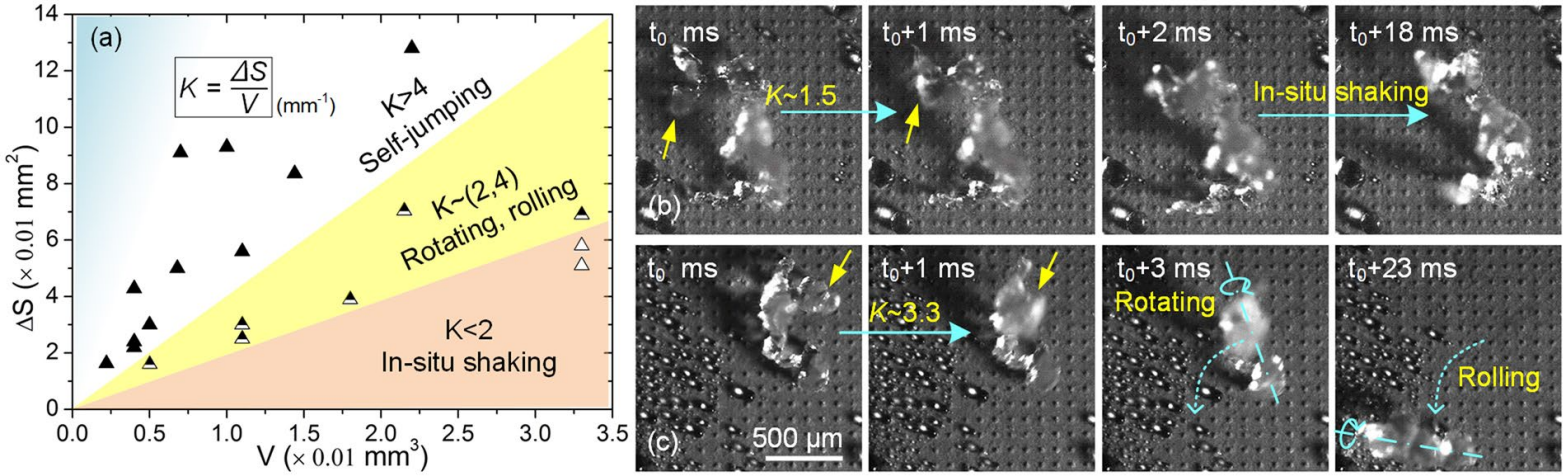
The motion states of the melting frost with different *K* values (*K* = ∆*S*/*V* (mm^−1^)). (**a**) The melting frost showed *in-situ* shaking when *K* < 2 mm^−1^, rolling and rotating (but no jumping) when 2 mm^−1^ < *K* < 4 mm^−1^, and obvious self-jumping when *K* > 4 mm^−1^; (**b**) the melting frost *in-situ* shaking when *K*~1.5 mm^−1^; (**c**) the melting frost rotating and rolling on NNS surface when *K*~3.3 mm^−1^.

Equation (2) also suggests that, the motion states of the melting frost are also influenced by its mass (or volume). And experiments show various motion states of melting frost after the self-triggered deformation: *in-situ* shaking, rotating, rolling, and self-jumping. Now that it is unable to obtain the mass and the transformed kinetic energy of the melting frost directly, we use the estimated volume (*V*) and the superficial area change per unit time (∆*S*) instead. To further discuss the relationship between the transformed kinetic energy and the volume, we conducted a large number of experiments and recorded the estimated volume and the superficial area change per unit time of every melting frost (see Fig. 4a). Here, we define a parameter *K* = ∆*S*/*V* (mm^−1^) to express the motion states of the melting frost: the melting frost showed only *in-situ* shaking when *K* < 2 mm^−1^ (see also Fig. 4b, *K*~1.5 mm^−1^), rolling and rotating (but not leaving the surface) when 2 mm^−1^ < *K* < 4 mm^−1^ (see also Fig. 4c, *K*~3.3 mm^−1^), and obvious self-jumping when *K* > 4 mm^−1^ (see also Fig. 2, *K*~9.3 mm^−1^). The melting frost with larger *K* values often shows more powerful movement, and is more likely to leave the surface. More interesting, we can see that large (or small) *K* values are often obtained from relatively small (or large) melting frost. Defrosting experiments also demonstrate that the frost thickness greatly influences the *K* values: thick frost is more likely to melt and shrink into a large water/ice mixture with small *K* values, and when the thickness of the frost reaches a certain value (ranging from about 1.5 mm to 2 mm), no self-jumping phenomena can be observed. (see Video S2).

**Figure 5:**
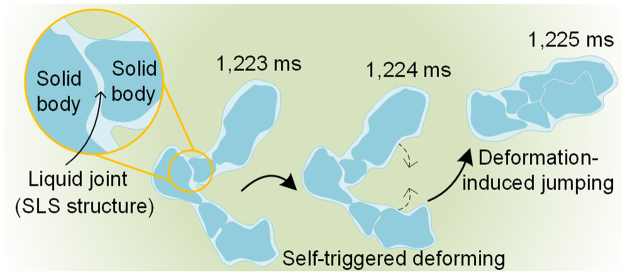
The schematic of irregular-shaped solid/liquid mixture deforming and self-jumping. After shrinking and splitting step, the melting frost developed into an L-shaped solid/liquid mixture. In the SLS structure, the liquid bridges worked as lubricants in hinge joints, so the self-triggered deforming was most likely to occur at the liquid joints. And after the self-triggered deforming at 1,224 ms, the two arms of the L-shaped mixture were merged into a larger one, with a relatively regular shape. At this moment, a great decrease of superficial area and a great increase of velocity occurred in less than 1 ms.

Consequently, the kinetic energy transformed from instantaneous superficial area change is the intrinsic reason for various melting frost self-propelled movements, and when the transformed energy reaches a certain amount (namely, *K* > 4 mm^−1^), the melting frost shows self-jumping phenomena. There is no doubt that sufficient transformed energy came from the instantaneous self-triggered deforming process. In other words, the self-triggered deforming at 1,224 ms was the “blasting fuse” for the self-jumping phenomenon. Then, why the instantaneous self-triggered deformation could happen? After shrinking and splitting step, the melting frost developed into an L-shaped solid/liquid mixture. In this mixture, two or more solid bodies (ice phase) were wrapped and chained together by melted liquid water. And between two solid bodies, a liquid bridge formed, working as lubricants in hinge joints (see Fig. 5). This solid body-liquid joint-solid body (SLS) structure was in a metastable state that was easy to be broken, because the water surface tension provides the liquid with the tendency to retract to a minimum superficial area. The liquid joint between two solid bodies had ultralow friction coefficient, making it easier for solid bodies to show deformation like joint movements. It was hard for solid bodies themselves to deform in such a short instant, so the self-triggered deforming was most likely to occur at the liquid joints. And after the self-triggered deforming at 1,224 ms, the two arms of the L-shaped mixture were merged into a larger one, with a relatively regular shape. At this moment, a great decrease of superficial area and a great increase of velocity occurred in less than 1 ms. In summary, the instantaneous self-triggered deformation released sufficient energy in a very short moment, directly leading to self-jumping of the melting frost, and the SLS structure in the irregular-shaped melting frost greatly promoted the instantaneous self-triggered deforming and the resulting self-jumping phenomena.

**Figure 6:**
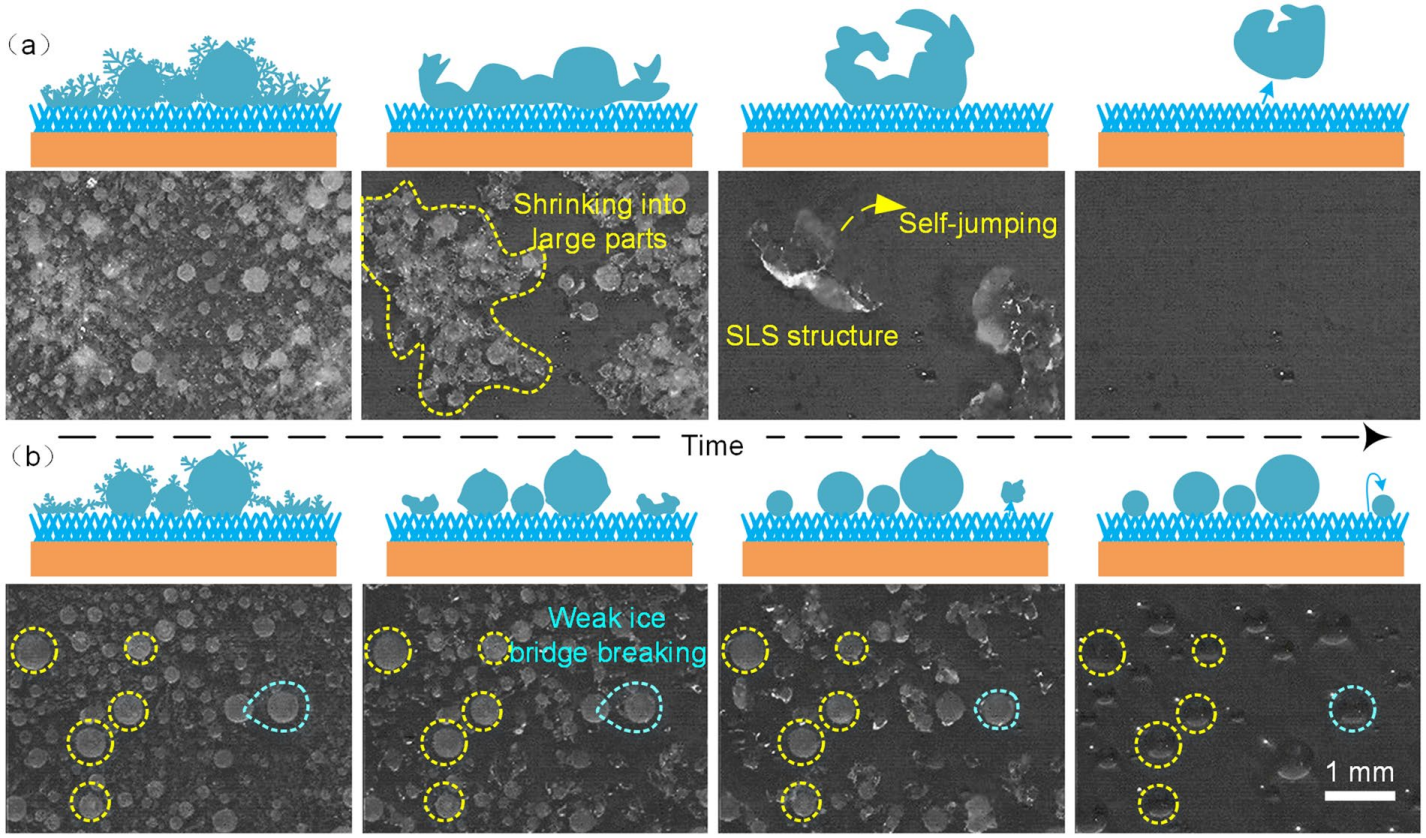
The schematic and experimental images of the frost with strong (**a**) weak (**b**) ice bridges between the frozen droplets. The frost with strong ice bridges could shrink into an irregular-shaped solid/liquid mixture, and then showed self-jumping phenomena, leaving a dry area. The frost with weak ice bridges broke into small near-spherical parts, which could not show self-jumping phenomena, leaving micro-droplets pinning on the surface after the defrosting process.

Then, how to obtain the SLS structure in the melting frost? Boreyko *et al*. reported that the frost propagated using interdrop ice bridge^9^. We also found different kinds of ice bridges between frozen droplets. However, not all frozen droplets could show self-jumping performance: some ice bridges just broke in the melting process, with no shrinking motion, leaving melted droplets pinned on the surface. Chu *et al*. also mentioned that the frost crystals on the droplet surface strengthened the contact between droplets effectively at the beginning of the melting and prevented their breaking up^22^. Here, we name “continuous frost” to describe the frost that has strong ice bridges between frozen droplets. For the purpose of defrosting, the frost should be continuous to provide enough attractive force for gathering micro-droplets into a larger one. A strong ice bridge could melt into a strong liquid water bridge in the melting process, attracting micro-droplets together into a larger, irregular-shaped solid/liquid mixture. In this mixture, the SLS structure took shape. When the metastable SLS structure was broken, the self-triggered deforming occurred and thus, provided enough surface energy change for self-jumping (see Fig. 6a). After the self-jumping phenomena, the surface in field-of-view became free of ice and water droplets. For a weak ice bridge, it may break in the melting process because it was hard to keep an ultrathin water bridge. Accordingly, the frost that are not “continuous” would show less self-jumping phenomena and more water droplets could remain pinning on the surface. As shown in Fig. 6b, the frozen droplets in yellow dash line did not show any movements in the defrosting process. So frost with strong ice bridges may be a necessary condition for SLS structure formation.

## Conclusions

The frosting and defrosting experiments were conducted on the nano-needle superhydrophobic surfaces. The melting frost dynamics on the prepared surfaces were studied and self-jumping phenomena of the melting frost were observed. The excellent superhydrophobicity of the nano-needle surface ensured ultralow adhesion between the melting frost and the surface. The kinetic energy transformed from instantaneous superficial area change in self-triggered deforming step is the intrinsic reason for various melting frost self-propelled movements, and when the transformed energy reached a certain amount (namely, *K* > 4 mm^−1^ in this work), the self-jumping phenomena occurred. The melting frost self-jumping was always accompanied by great instantaneous self-triggered deformation, which caused great surface energy release in a very short moment. We also found the facilitating conditions for melting frost self-jumping phenomena: the solid body-liquid joint-solid body (SLS) structure in the irregular-shaped melting frost which promoted the instantaneous self-triggered deforming and the resulting self-jumping phenomena, and the strong ice bridges in the frost which provided a prerequisite for SLS structure formation. With the help of melting frost self-jumping performance, the sample surfaces can evidently shorten the defrosting process and reduce, even avoid residual droplets after defrosting. The present study will provide an efficient way for defrosting applications or an inspiration for further research on high-efficiency defrosting.

## Methods

### Preparation of Nano-Needle Superhydrophobic surface

The nano-needle superhydrophobic surface with micro-pillar was prepared by an electrochemical method. First, the micro-pillar array was fabricated on a copper foil (99.95%, 3 × 2.5 × 0.5 cm^3^) by a lithography-assisted chemical etching in a mixed aqueous solution of 1 M FeCl_3_ and 1 M HCl. The diameter and the height of the pillar are ~40 µm and ~10 µm, respectively. And the central distance of the adjacent pillars is ~100 µm. Then, the Cu(OH)_2_ nano-needles were obtained by electrolysis and chemical deposition in a 1 M NaOH solution for 10 min under a current density of 2.5 mA cm^−2^ followed by FAS-17 modification^33^.

### Frosting and Defrosting Experiments

The frosting and defrosting experiments were conducted in a transparent PMMA chamber using a water-cooled Peltier cooling stage, the temperature of which was feedback-controlled by a thermometer. And the humidity of the experiment environment was controlled by pumping humid air at a certain rate into the chamber. The NNS sample surfaces were placed on the Peltier cooling stage horizontally, to simulate the mild frosting conditions, such as the frosting environment of air conditioners.

The defrosting performance was *in situ* visualized and recorded using a high-speed camera (Olympus i-speed LT) at 1,000 fps. The room temperature and room relative humidity were 22 ± 1 °C and 15 ± 5%, respectively. In this process, the NNS samples were firstly cooled down in the humid environment and the frost continuously accreted. When the frost accreted to a certain amount, the cooling stage was powered off and the samples were warmed up to room temperature naturally. The dynamic melting performance of the frost were recorded.

### Data Availability

The datasets generated or analysed during the current study are available from the corresponding author on reasonable request.

## Electronic supplementary material

Supplementary Information

Supplementary Video S1

Supplementary Video S2
