# Supplementary material for: Self-jumping Mechanism of Melting Frost on Superhydrophobic Surfaces
Source: Sci Rep. 2017 Nov 7;7:14722. doi: 10.1038/s41598-017-15130-0 (PMC5676707; doi:10.1038/s41598-017-15130-0)
Supplement: Supplementary file 1 — Supplementary Information [file 41598_2017_15130_MOESM1_ESM.pdf]

Supplementary Information

Self-jumping Mechanism of Melting Frost on Superhydrophobic Surfaces

Xiaolin Liu, Huawei Chen,* Zehui Zhao, Yamei Wang, Hong Liu and Deyuan Zhang

*School of Mechanical Engineering and Automation, Beihang University, Beijing, 100191, China. *Email: chenhw75@buaa.edu.cn*

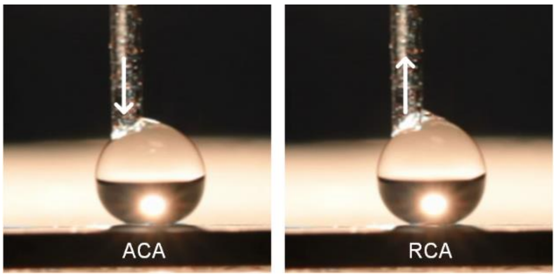

Figure S1. The advancing contact angle (ACA) and the receding contact angle (RCA) of the nano-needle superhydrophobic surface. The ACA and RCA were 170.3 ± 0.8° and 169.4 ± 1.0°, respectively. Thus, the contact angle hysteresis was 0.9 ± 0.5°, demonstrating the excellent superhydrophobicity of the prepared surface.

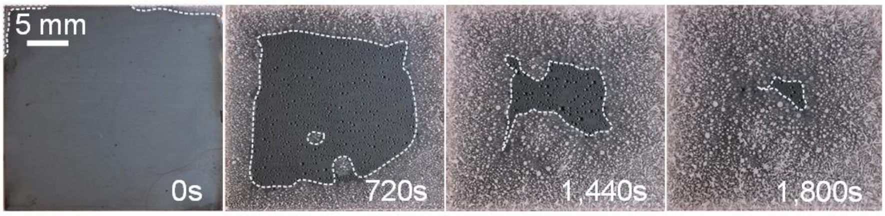

Figure S2. Frost propagation on the nano-needle superhydrophobic surface. The sample surface was fully covered by frost in 1,980s, showing excellent frost-delaying performance.

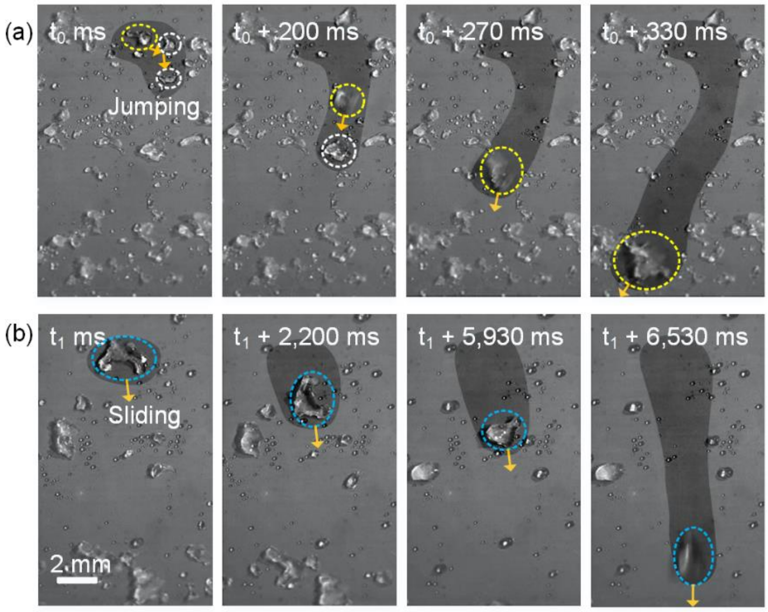

Figure S3. The duration for a jumping melting frost (a) and a sliding melting frost (b) leaving the sample surface. The snapshots were taken on the same surface that was set at an inclination angle of 45º. It took a jumping melting frost just 330 ms to leave the sample surface, and in this progress the jumping melting frost (highlighted by yellow dashed circles) triggered the neighboring melting frost (highlighted by white dashed circle) and evidently accelerated the defrosting process. However, the time cost for a sliding melting frost (highlighted by blue dashed circle) was too much longer, reaching 6,530 ms. The results demonstrated the superiority of the self-jumping phenomena over other self-propelled movements, in terms of shortening the defrosting process.

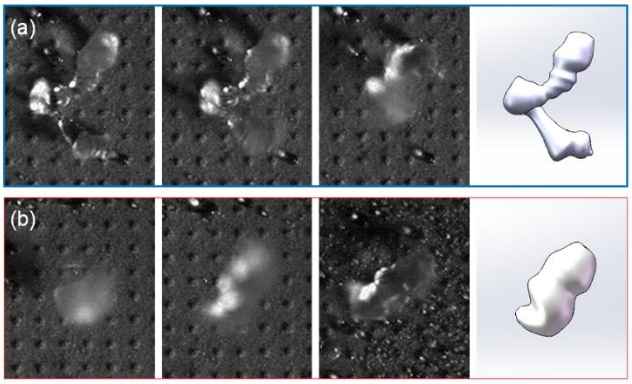

Figure S4. The illustration of "three-dimensional reconstruction" method. For every piece of melting frost, we took more than three pictures of the jumping frost from different angles, to roughly figure out its 3D shape. A three-dimensional modeling software "Solidworks 2013" was used to build the 3D model of the melting frost, and then its volume and superficial area can be automatically measured by this software. We built two 3D models for every melting frost before and after the instantaneous deformation, and obtained the volume change and the superficial area change.

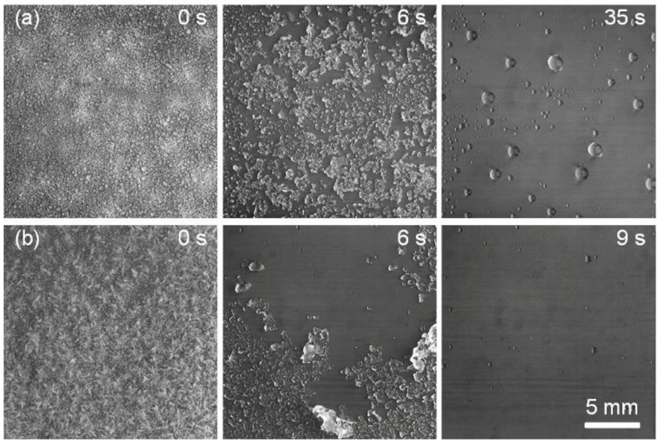

Figure S5. The influence of airflow rate on frost detaching from the surface. (a) Frost melted without airflow; (b) frost melted with a horizontal airflow (the

temperature of the airflow was 25°C, and the flow rate was 2.5 m/s).

With a horizontal airflow (the temperature of the airflow was 25°C, and the flow rate was 2.5 m/s), the frost melting process was accelerated and the frost jumping was promoted. The time cost for frost melting was decreased from 35s to 10s, and the frost jumping phenomena were more obvious. The results are reasonable and predictable. The warm airflow (25°C), working as a heat source, enhanced the heat transfer of the frost, and thus accelerated the melting process. Moreover, the airflow also improved the deformation of the melting frost and provided an initial horizontal velocity, and thus promoted the frost jumping and detaching the surface.

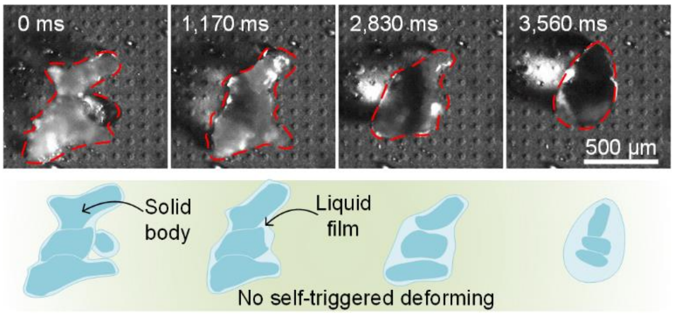

Figure S6. An irregular-shaped melting frost without self-jumping phenomena. Although the melting frost had apparent irregular shape, it melted very slowly and showed unobservable movements. The reason was that the long edges of the solid bodies were jointed relatively in balance by liquid films, with no self-triggered deformation. The metastable liquid joints in the SLS structure played a crucial role for self-triggered deformation.

Video S1. The dynamic defrosting process of the nano-needle superhydrophobic surface. This process was recorded using a high-speed camera (Olympus i-speed LT) at 1,000 fps and played at 30 fps. The defrosting process could be mainly divided into three steps: melting frost shrinking and splitting, instantaneous self-triggered deforming and deformation-induced jumping.

Video S2. The melting frost showing no self-jumping phenomenon when the initial thickness reached 1.5 mm. The melting frost could only show movements like in-situ shaking.
